# Quantitative Determination of Common Urinary Odorants and Their Glucuronide Conjugates in Human Urine

**DOI:** 10.3390/metabo3030637

**Published:** 2013-08-07

**Authors:** Maria Wagenstaller, Andrea Buettner

**Affiliations:** 1 Department for Chemistry and Pharmacy, University of Erlangen-Nuremberg, Emil Fischer Center, Schuhstr. 19, Erlangen 91052, Germany; E-Mail: maria.wagenstaller@fau.de; 2 Fraunhofer Institute for Process Engineering and Packaging (IVV), Giggenhauserstr. 35, Freising 85354, Germany

**Keywords:** metabolization, excretion, volatile, stable isotope dilution assay, two-dimensional high resolution gas chromatography, mass spectrometry, quantification, glucuronide, glucuronidase assay, biotransformation

## Abstract

Our previous study on the identification of common odorants and their conjugates in human urine demonstrated that this substance fraction is a little-understood but nonetheless a promising medium for analysis and diagnostics in this easily accessible physiological medium. Smell as an indicator for diseases, or volatile excretion in the course of dietary processes bares high potential for a series of physiological insights. Still, little is known today about the quantitative composition of odorous or volatile targets, as well as their non-volatile conjugates, both with regard to their common occurrence in urine of healthy subjects, as well as in that of individuals suffering from diseases or other physiological misbalancing. Accordingly, the aim of our study was to develop a highly sensitive and selective approach to determine the common quantitative composition of selected odorant markers in healthy human subjects, as well as their corresponding glucuronide conjugates. We used one- and two-dimensional high resolution gas chromatography-mass spectrometry in combination with stable isotope dilution assays to quantify commonly occurring and potent odorants in human urine. The studies were carried out on both native urine and on urine that had been treated by glucuronidase assays, with analysis of the liberated odor-active compounds using the same techniques. Analytical data are discussed with regard to their potential translation as future diagnostic tool.

## 1. Introduction

Human urine contains an abundance of important information for physicians. The diagnostic potential of urine composition is mirrored in a broad range of assays, for example testing for glycosuria (etiologies include diabetes mellitus, liver and pancreatic disease, Fanconi’s syndrome, and Cushing’s syndrome), ketonuria (most commonly associated with uncontrolled diabetes), and testing for nitrites (associated with urinary tract infections) among many others [1]. However, with regard to its volatile and odorous profile, human urine has not yet been sufficiently evaluated. Nevertheless, some researchers (mainly in the 1960’s and 1970’s) tried to reveal the diagnostic potential of the volatile profile of human urine, and in several cases observed distinct differences between healthy and diseased individuals. Liebich and Zlatkis, for example, found that urinary volatile profiles were different in patients suffering from diabetes mellitus compared to healthy volunteers [2,3,4,5]. Still, few efforts have been paid in subsequent years to characterize the odor profile and the odorants in human urine. Recently, the characteristic odorant spectrum of human urine has been explored by gas chromatography-olfactometry (GC-O) [6]. Compounds were elucidated by means of a combinatory approach using human-sensory and chemo-analytical techniques such as two-dimensional GC-O/mass spectrometry; however, quantifications were not accomplished.

Moreover, it is known that the concentrations of some volatiles in urine can vary depending on the hormonal status of the donor [7]. This has been shown in the case of acetone [7]. Accordingly, quantitative data on volatiles would help to find out if similar effects for example caused by hormonal status also exist for other compounds.

As mentioned before, the urine of diseased people often contains compounds that are not present—or are in different amounts—in the urine of healthy people. In some cases, even the sensory impressions of the urine of diseased people can be quite divergent from that of healthy individuals as has been reported in a range of cases: The urine of patients suffering from maple syrup urine disease has a maple syrup-like odor [8,9], while the urine of tyrosinemia patients smells rancid [9] , and the urine of people suffering from trimethylaminuria has a fishy odor [9,10], just to name some examples.

With the emergence of metabolomics and biomarkers research urine composition became even more interesting in the last years. Among other things, some urinary volatile compounds have been proposed as biomarkers for prostate cancer [11,12] and breast cancer [13].

The fact that urinary odor may contain important information for cancer diagnosis is mirrored by the attempt to train dogs to sniff specific associated smells. Studies revealed that canines were able to distinguish between urine of bladder cancer patients and urine of healthy subjects [14,15].

Furthermore, it was possible to discriminate between healthy humans and tuberculosis patients by analysis of the volatiles in human urine based on identification and quantification experiments [16]. It was also attempted to diagnose urinary tract infections by detecting volatiles with an electronic nose [17].

To date, only certain diseases can be diagnosed by detection of elevated concentrations of specific (volatile) compounds in human urine, specifically trimethylamine and trimethylamine N-oxide in trimethylaminuria [10]. Also, some volatiles can be used to obtain information about a person´s lifestyle, for example their smoking behavior. Elevated concentrations of acetonitrile have been found in the urine of recent smokers (active as well as passive smokers) [18].

Apart from its use in diagnostics, human urine can also potentially provide useful information with regard to nutrition. Characterization of odorants or their metabolites in human urine may increase our understanding of the fate of different dietary constituents.

Still, it is a well-known phenomenon that urinary smell can be affected by nutrition. A prominent example for this is the specific smell that may be associated with the consumption of asparagus, being often described as rotten cabbage-like [19,20]. Investigations showed that this specific smell is induced by metabolization of asparagusic acid and its derivatives [19]. Moreover, the smell does only occur in some individuals; the genetic basis for this observation is yet unknown [20].

Apart from a few examples, metabolization and excretion of volatiles originating from dietary sources has barely been studied in humans. One of the rare examples is the excretion of 5-hydroxymethyl-2-furoic acid in human urine after the consumption of 5-hydroxymethylfurfural-containing food like coffee, dried fruit, honey and alcoholic beverages [21]. Other examples are the urinary excretion of the glucuronide of 4-hydroxy-2,5-dimethyl-3 (2*H*) furanone as the major metabolite of 4-hydroxy-2,5-dimethyl-3 (2*H*) furanone after the consumption of strawberries [22] and the excretion of the estragole metabolites 1′-hydroxyestragole, 1′-hydroxyestragole glucuronide and p-allylphenol glucuronide in urine of humans consuming fennel tea [23].

Apart from that, work focusing on common odorous compounds in human urine hardly exists.

With regard to the latter aspect, it is essential to know in which concentration ranges odorants are commonly present in the urine of healthy humans in order to be able to detect abnormalities resulting from diseases, environmental influences or diets.

Thus, the first aim of the present study was to quantify selected common odorants in human urine of healthy people who were allowed to consume their freely chosen meals.

In the second part of the study, investigations were also targeted at conjugated derivatives of common odorants in human urine. Glucuronidation is an important pathway among the phase II reactions to eliminate lipophilic xenobiotics as well as endobiotics by rendering them more hydrophilic [24]. The resulting glucuronides are mainly excreted by the kidneys. In most cases, the resulting glucuronides are chemically and biologically less active than the original compounds. Accordingly, the formation of glucuronides is commonly an important step with regard to detoxification reactions [24]. However, there is increasing evidence that in some cases glucuronide conjugates might be formed which also represent a physiologically active form. A prominent example is morphine: The morphine-6-glucuronide is even more analgesic than morphine itself [25,26].

As a logical consequence, some glucuronides are also used as a diagnostic means. For example, ethyl glucuronide in urine is used as a marker to control the success of alcohol dehabituation [27,28].

In this context it needs to be kept in mind that for some drugs the rate of glucuronidation might be different between males and females [29,30]. Other factors affecting glucuronidation of specific drugs are age, weight, cigarette smoking, ethnicity, diet, coadministered drugs, genetic factors, hormonal factor, and certain diseases (*i.e.*, HIV, hypothyroidism, and fulminant hepatitis) [29,30]. Whether the same is true for other compounds than these drugs, e.g., for dietary constituents, is also not yet comprehensively addressed.

Commonly, glucuronides represent the major part of metabolites of many phenols, alcohols and carboxylic acids [31]. Thereby, endogenous substrates for glucuronidation are for example steroids, bile acids, bilirubin and retinoids [32,33]. Exogenous substrates are, *inter alia*, drugs, environmental pollutants or food constituents [34,35].

The knowledge about the formation and excretion of glucuronides is mainly based on drugs with a known pharmacological effect, however studies on metabolization of other compounds, for example volatiles, hardly exist. In our recent study on odorants in human urine we also targeted our identification experiments on common glucuronide conjugates of odorants [6]. However, no quantification of the target substances was performed in this study. Thus, the amount of glucuronide-formation as well as inter-individual differences in the extent of formation of glucuronides remained unclear.

Consequently, the second aim of the present study was to quantify selected common odorant-glucuronide conjugates in human urine of healthy subjects. Thereby, the aim was to compare the concentrations of the respective glucuronide conjugates to those of the free odorants.

Such insights are the basis for future studies targeting potential deviations from the common status.

## 2. Results

All urine samples showed negative results for bilirubin, ketones, protein, blood, nitrite and leucocytes in the dipstick-testings. Furthermore, all urine samples had normal amounts of urobilinogen and glucose. pHs were between 5.0 and 6.5, specific gravity/density between 1.000 and 1.030.

As none of the urine samples had any abnormal values, all samples could, consequently, be included in our experiments.

### 2.1. Quantification of Odorants in Native Human Urine

**Figure 1:**
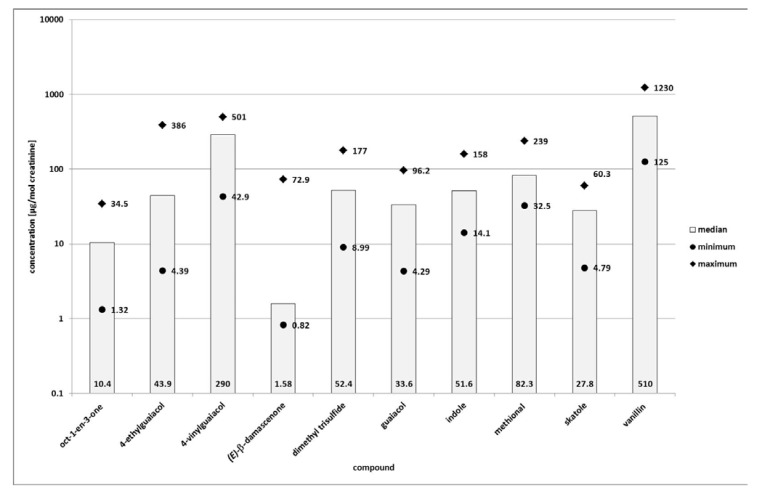
Minimum, median and maximum concentrations of selected odorants in native human urine. Concentrations are given in [µg/mol creatinine] and displayed on a logarithmic scale.

Based on our previous odor identification experiments in human urine [6], we selected 10 characteristic compounds for quantification. Among these were alcohols, phenols, ketones, aldehydes, pyrroles as well as a dialkyl trisulfide. Main consideration for the selection was that these compounds represent different substance classes as well as different chemical reactivities, volatilities, and polarities. Quantification experiments revealed that all compounds were present in median concentrations between 0.02 and 2.55 µg/L corresponding to values between 1.58 and 510 µg/mol creatinine. The lowest substance concentrations were found for the two ketones oct-1-en-3-one and *(E)*-β-damascenone, with median concentrations in the ng/L range. The highest concentrations were found for two phenols, 4-vinylguaiacol and vanillin, with median concentrations of 1.06 µg/L and 2.55 µg/L, respectively. The median concentrations of the remaining compounds 4-ethylguaiacol, dimethyl trisulfide, guaiacol, indole, methional, and skatole were between 0.10 µg/L and 0.51 µg/kg, or values between 28 and 82 µg/mol creatinine, respectively (Figure 1 and online supplementary Table S1). Generally, creatinine concentrations were between 1,923 and 21,370 µmol/L.

Pronounced inter-individual variations were observed for several compounds. The highest variation was found for *(E)*-β-damascenone, for which the maximum concentration was 89-times higher than the lowest concentration measured (irrespective if calculated on basis of the concentrations in µg/L or on basis of concentrations normalized by creatinine concentrations (in µg/mol creatinine). Also, when inter-individual variations were calculated on basis of the non-normalized values (in µg/L), we found some additional major inter-individual variations for 4-ethylguaiacol, dimethyl trisulfide, and guaiacol, with factors of 33, 13 and 12, respectively (maximum concentration divided by minimum concentration). In contrast to these high variations, the concentrations of oct-1-en-3-one, skatole, indole, methional, 4-vinylguaiacol, and vanillin varied only by factors 3 to 5 (maximum concentration divided by minimum concentration). When calculation of the inter-individual variation was based on the data normalized by creatinine concentrations, we observed even larger inter-individual variations. The variations for 4-ethylguaiacol were then nearly as high as for *(E)*-β-damascenone with a factor of 88 between the maximum and the minimum concentrations. High variations were also observed for dimethyl trisulfide, guaiacol, and oct-1-en-3-one with factors between 20 and 26. For 4-vinylguaiacol, indole, skatole, and vanillin, the factors were about 10. The least pronounced inter-individual variation was found for methional, still being considerable with a factor of 7.

Apart from that, a difference between urine of male test persons and urine of female test persons was observable. The median concentration of most compounds was higher in urine of females than in urine of males (see online supplementary material Figure S1). This was true for oct-1-en-3-one, 4-ethylguaiacol, 4-vinylguaiacol, dimethyl trisulfide, guaiacol, indole, methional, skatole, and vanillin (concentration normalized to µg/mol creatinine). Only the concentration of *(E)*-β-damascenone was higher in urine of male test persons (concentration normalized to µg/mol creatinine). The observed differences were especially striking for indole, 4-vinylguaiacol and vanillin: for these compounds even the minimum concentrations found in urine of females were higher than the corresponding maximum concentrations in urine of males, resulting in the fact that there was no overlapping of concentration ranges in male and female urine. Still, when regarding these differences on a statistical basis, the results were only significant for vanillin (Mann-Whitney-test at *p* = 0.05). Nevertheless, at *p* = 0.1, we found some additional significant differences for skatole, methional, and indole, while the differences were not significant for dimethyl trisulfide and 4-ethylguaiacol (Mann-Whitney-test at *p* = 0.1). The Mann-Whitney-test could not be performed for oct-1-en-3-one, 4-vinylguaiacol, *(E)*-β-damascenone, and guaiacol because the sample sizes were too small in these cases. The sample sizes were too small for these compounds as they were in some samples present below their limit of quantification and in some cases even below their limit of detection.

Online supplementary material Table S1 shows the determined concentrations of odorants in native human urine, an additional table given in online supplementary material Table S2 provides a compilation of standard compounds, isotopic labeled standards, their mass, and the m/z-ratios selected for quantification.

### 2.2. Quantification of Odorants in Enzymatically Hydrolyzed Human Urine

#### 2.2.1. Addition of Sodium Azide

The addition of sodium azide to the urine before incubation with glucuronidase revealed that the concentrations of the compounds selected for our experiments remained stable regardless of addition of sodium azide as was ensured by additional quantitative experiments.

#### 2.2.2. Quantification of Selected Odorants in Glucuronidase-Treated Human Urine

Based on our previous odor identification experiments in glucuronidase-treated human urine [6], we selected 12 compounds for quantification. The selected odorants were 3-methylbutanoic acid, 4-ethylguaiacol, 4-vinylguaiacol, *(E)*-β-damascenone, butanoic acid, dimethyl trisulfide, guaiacol, indole, methional, skatole, sotolone, and vanillin. As mentioned above, the compounds were selected to represent different substance classes as well as different chemical reactivities, volatilities, and polarities. 4-Ethylguaiacol, 4-vinylguaiacol, *(E)*-β-damascenone, dimethyl trisulfide, guaiacol, indole, methional, skatole, and vanillin were also quantified in the native samples (see above). Oct-1-en-3-one, which was quantified in the native samples, could not be quantified in the hydrolyzed samples hence it was near the limit of detection and in contrast to the native samples, oct-1-en-3-one was additionally superimposed by other substances in the hydrolyzed samples. Therefore, a quantification was not possible. Limits of quantification of all compounds were depending on the compound, sample pretreatment (native or hydrolyzed) and preparation of calibration line. Three compounds were additionally quantified in the glucuronidase-treated samples. These were 3-methylbutanoic acid, butanoic acid, and sotolone. Butanoic acid and 3-methylbutanoic acid were previously found to be not important odorants in native human urine (sotolone was even below the limit of quantification in the native samples), but they were obviously released to a major extent in glucuronidase-treated human urine [6].

**Figure 2:**
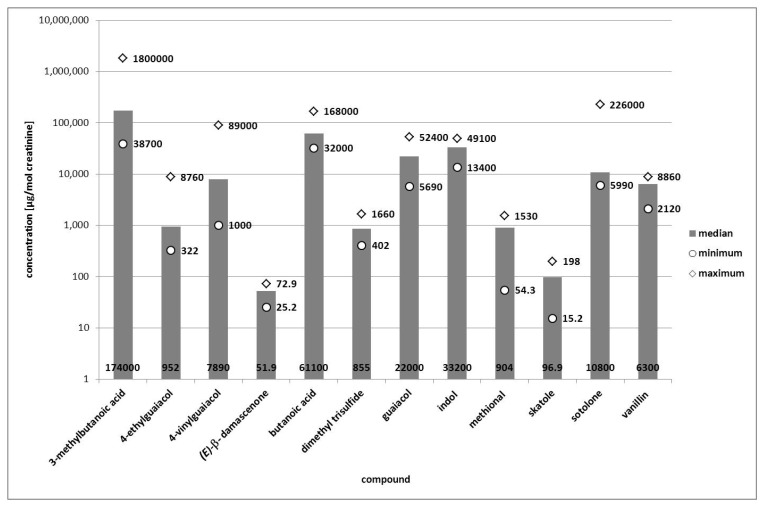
Minimum, median and maximum concentrations of selected odorants in glucuronidase-treated human urine. Concentrations are given in [µg/mol creatinine] and displayed on a logarithmic scale.

Generally, the quantified compounds were present in a broad range of concentrations, namely in median concentrations between 0.39 and 1,050 µg/L (online supplementary material Table S3), corresponding to median concentrations between 52 and 174,000 µg/mol creatinine (online supplementary material Table S3 and Figure 2).

The creatinine concentrations of the samples were between 1,923 and 21,370 µmol/L (see Section 2.1) as aliquots of the same samples used for quantification in native urine were used for quantification in hydrolyzed urine. The lowest median concentrations were found for *(E)*-β-damascenone and skatole, which were both present in median concentrations below 1 µg/L. Dimethyl trisulfide, methional, and 4-ethylguaiacol were found in median concentrations between 1 and 10 µg/L, while vanillin, 4-vinylguaiacol and sotolone were present in median concentrations between 10 and 100 µg/L. The remaining four compounds guaiacol, indole, and the two acids butanoic acid and 3-methylbutanoic acid were found in median concentrations above 100 µg/L (online supplementary material Table S3 and Figure 2).

Again, major inter-individual variations were observed for several compounds. Thereby, the inter-individual variations were higher for most compounds when being compared on basis of the concentrations normalized by creatinine than when compared on basis of the non-normalized values. Only for indole and guaiacol it proved to be vice versa.

When compared on basis of the non-normalized values in [µg/L], the inter-individual variations were least pronounced for *(E)*-β-damascenone, dimethyl trisulfide, and vanillin (all factor 2 for maximum concentration divided by minimum concentration) and for butanoic acid and skatole (both factor 3). Two other compounds showed factors below 10: methional (factor 7) and indole (factor 8) when considering the non-normalized concentrations. Guaiacol, 4-vinylguaiacol, sotolone, and 3-methylbutanoic acid showed factors between 10 and 17. The most pronounced inter-individual variation was found for 4-ethylguaiacol. The maximum concentration found was 22-times higher than the minimum concentration.

As mentioned above, the variations were mostly higher when comparing on basis of the concentrations normalized by creatinine. The following compounds had factors lower than 10 when dividing maximum concentration by minimum concentration on basis of the concentrations normalized by creatinine: *(E)*-β-damascenone (factor 3), indole, dimethyl trisulfide, vanillin (all three factor 4), butanoic acid (factor 5), and guaiacol (factor 9). The remaining compounds had factors between 10 and 100: skatole (factor 13), 4-ethylguaicol (factor 27), methional (factor 28), sotolone (factor 38), 3-methylbutanoic acid (factor 47), and 4-vinylguaiacol (factor 89).

The differences between urine of male and urine of female probands were not as pronounced as in the native urine. No definitive trend was visible for most substances, as for some compounds the median concentration was higher in female urine than in male urine and for other compounds it was the other way around. All in all, the median concentration in [µg/mol creatinine] differed no more than factor 0.4 to factor 1.8 when dividing the median concentration in male urine by the median concentration in female urine (see also online supplementary material Figure S2). These relatively small variations between men and women are also reflected when performing statistics on the data. The variations were found to be not significant even at a p-value of 0.1 for guaiacol, methional, vanillin, skatole, 4-vinylguaiacol, 3-methylbutanoic acid, butanoic acid, indole, and dimethyl trisulfide when analyzing the data by Mann-Whitney-test. The Mann-Whitney-test could not be performed for 4-ethylguaiacol, sotolone, and *(E)*-β-damascenone, because the sample sizes were too small. The reason for the partly too small sample sizes is as explained above for the native samples that some compounds were in some samples present below their limit of quantification and in some cases even below their limit of detection.

For additional information on internal standards, ions selected for quantification and chemical information on the odorants quantified in these experiments, see online supplementary material Table S4.

## 3. Discussion

Our manuscript provides for the first time detailed quantitative data on common odorants in human urine, as well as of their respective conjugates. The results of the quantification experiments are provided both in the original concentrations [µg/L] as well as in concentrations normalized by creatinine concentration of the samples [µg/mol creatinine]. Normalization by creatinine concentration was done to eliminate the impact of diuresis on the concentration, representing a parameter that is generally accepted for this purpose [9].

Overall, the lowest concentrations in native urine were found for *(E)*-β-damascenone and oct-1-en-3-one. Nevertheless, those two substances also have lower odor thresholds in water than all the other compounds quantified in our experiments, with reported odor thresholds of 0.00075–10 µg/L for *(E)*-β-damascenone [36,37,38,39,40] and 0.0050–4.0 µg/L for oct-1-en-3-one [37,41,42,43,44]. That means that although present in low concentrations these compounds may still have an impact on the overall odor impressions of the samples, and might serve, accordingly, as diagnostic means. Interestingly, we found the largest inter-individual variations for *(E)*-β-damascenone, a compound, which is an important odorant in some foods. According to [45], *(E)*-β-damascenone is an important odorant in apple, coffee, wine, black tea, beer, honey and tomatoes. When looking at the dietary records of our test persons, we found out that the person, whose urine had by far the highest concentration of *(E)*-β-damascenone (73 µg/mol creatinine), drank 200 mL of black tea one hour before sample collection. None of the other test persons consumed *(E)*-β-damascenone containing food during such a short interval before sample collection. So this observation might serve as a case report on dietary influences on the odorant composition of human urine. Nevertheless, to support such assumptions, further detailed studies with controlled dietary interventions are needed as done recently in our group involving, e.g., a coffee intervention [46]. 

Apart from that, most compounds were found in higher concentrations in native urine of females than of males. This observation might serve as a hint that there is a gender specific difference in the metabolization or excretion of these odorants. Gender-related differences in metabolization have previously been described: For a series of drugs, the rate of glucuronidation has been found to be lower in females [29,30]. On the other hand, the differences might be also related to gender-specific differences in dietary habits as reported several times [47,48,49]. Interestingly, the sex-specific differences between the concentrations of the odorants in our experiments were not as pronounced in the enzymatically hydrolyzed samples as in the native samples.

Besides, to ensure that no bacterial degradations occurred during the deglucuronidation experiments, additional tests with addition of preservative were carried out, revealing that no quantitative changes occurred in comparison to incubated samples without preservative during 15 h of incubation at 37 °C. Additionally, one needs to keep in mind that other factors may contribute to an increase of the concentration of specific volatiles after the enzyme treatment. One of these factors is the additional sulfatase activity of the glucuronidase (see Section 2.1). Another factor is a potential aging effect of the urine during 15 h incubation time. Aging effects of urine after a storage time of five days in an open container have been described by Troccaz *et al.* [50]. As we were concerned that a 15 h incubation time could possibly lead to the liberation of compounds which were not derived from the liberation of glucuronidated compounds but from the aging of the urine, we conducted an additional experiment. We took two aliquots (each 10 mL) of the same urine sample and subjected one aliquot to the procedure described in 4.6.2 (enzymatic hydrolysis). The second aliquot was treated analogously but no enzyme was added. Then we performed a gas chromatography-olfactometry analysis as described in [6] on both samples. If the compounds found after enzymatic hydrolysis were indeed formed by an aging process, we would also have found them in the second aliquot of the sample. As this was not the case, we concluded that the aging effect was of minor importance for the conditions used in our experiments.

Glucuronides are the vast majority of phase II-conjugates. In the literature, there were no previous reports on quantitative data for most of the compounds quantified in our experiments, with the only exceptions for some phenols, namely vanillin, guaiacol and 4-ethylguaiacol, and for butanoic acid. These three phenols had been quantified before, as they have been assumed to be biomarkers of wood smoke exposure [51,52,53]. Still, these compounds were only quantified in hydrolyzed and not in native urine, so that no conclusions can be drawn about the presence and quantities of the respective free or de-conjugated compounds. The mean urinary concentrations of non-exposed subjects were 710 µg/L [52], 691 µg/L [51], 720 µg/L [54], 38800 µg/mol creatinine [53] for guaiacol, 60 µg/L [52] for 4-ethylguaiacol, and 70 µg/L [52], 57 µ/L [51], 4638 µg/mol creatinine [53] for vanillin. [51] and [53] reported a mean concentration of ethylguaiacol (without specification of the 4-substituation of the ethyl-moiety) with 25 µg/L [51] and 3846 µg/mol creatinine [53]. With regard to our data, we decided to calculate and discuss the median concentrations as the inter-individual variations were in several cases quite pronounced. In order to be able to compare our results to those of Dills, Neitzel and Bieniek, we additionally calculated the mean concentrations for the three phenols. In our experiments, the mean concentration of guaiacol was 154 µg/L (26,900 µg/mol creatinine), of 4-ethylguaiacol 20 µg/L (2,330 µg/mol creatinine) and of vanillin 33 µg/L (5,790 µg/mol creatinine). Comparing these concentrations, one can conclude that the mean concentrations of guaiacol and 4-ethylguaiacol were lower in our experiments compared to those of [51,52,53,54]. The mean concentration of vanillin in our experiments was lower than the values determined by [51,52], but higher than those determined by [53]. However, all these differences are of minor importance, considering the fact that huge inter-individual variations were observed in our experiments as well as in the previous studies of the other researchers [51,52,53,54]. Interestingly, Dills *et al.* and Neitzel *et al.* [51,52,53] conducted an acidic hydrolysis, while we and Bieniek *et al.* [54] chose to conduct an enzymatic hydrolysis. The enzymatic hydrolysis is more specific for the glucuronides (though the glucuronidase in our experiments contained also sulphatase activity). Therefore, the slightly lower concentrations in our experiments could be an indication that the hydrolysis was more gentle. The only compound, apart from those three phenols, for which quantitative data existed, is butanoic acid. Chalmers *et al.* [55] found butanoic acid in concentrations between 0 and 63 mg/g creatinine which is 0 and 7,100,000 µg/mol creatinine, while Perry *et al.* [56] determined this compound in concentrations between 0 and 0.26 µg/mg creatinine corresponding to 0 and 29,000 µg/mol creatinine. Our results lay well between these values with concentrations between 32,000 µg/mol creatinine and 168,000 µg/mol creatinine in the hydrolyzed samples (median 61,100 µg/mol creatinine).

With regard to methylbutanoic acid, no distinction was made between the 2- and 3-isomers in previous investigations [6]. In the present study, differentiation between 2- and 3-methylbutanoic acid was accomplished based on the obtained chromatographic and mass spectrometric data, leading to the observation that 3-methylbutanoic acid is the predominant isomer. With regard to sensory or diagnostic relevance this observation is, nevertheless, of minor importance as both isomers have been reported with similar odor thresholds as well as comparable odor qualities.

**Figure 3:**
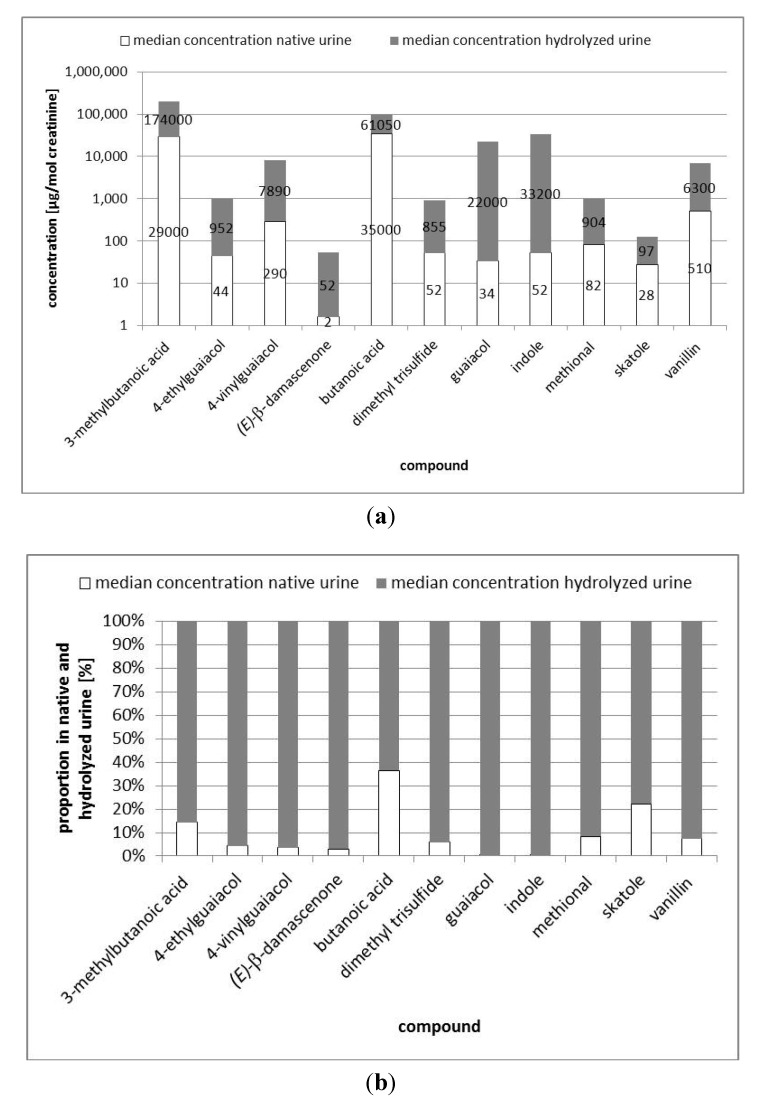
(**a**) Comparison of median concentrations of selected odorants in native and glucuronidase-treated human urine. Concentrations are given in [µg/mol creatinine] and displayed in a logarithmic scale. (**b**) Comparison of median concentrations of selected odorants in native and glucuronidase-treated human urine. Concentrations are given in percent, the concentration of the compounds in the glucuronidase-treated urine is set 100 percent.

Comparing the odorant concentrations in the native urine samples to those in the enzymatically hydrolyzed samples, a dramatic increase was observed for most compounds after hydrolysis. Apart from skatole, the median concentration (normalized by creatinine) of which only increased by a factor of 3 after hydrolysis, the median concentrations of all remaining compounds increased by at least a factor 11 (Table 1). Thereby, the concentrations of methional, vanillin, dimethyl trisulfide, 4-ethylguaiacol, 4-vinylguaiacol, and *(E)*-β-damascenone increased by factors between 11 and 33 after enzymatic hydrolysis (Table 1), meaning that the major part of these odorants is excreted as glucuronides than as the original compounds. However, the most extreme increases were observed for indole and guaiacol, with factors of 638 and 647 (median concentration in hydrolyzed urine divided by median concentration in native urine). Figure 3a displays the amounts of selected compounds present in their free form as well as the amounts of glucuronide conjugates of the respective compounds in logarithmic scales. In Figure 3b, emphasis is further placed on the ratios of the free to their glucuronidated substances, thereby displaying the respective concentrations on a percentage basis.

**Table 1:** Comparison of concentrations of odorants in native and glucuronidase-treated human urine.

No. ^1,2^	Compound ^3^	Median concentration native urine [µg/mol creatinine]	Median concentration glucuronidase-treated urine [µg/mol creatinine]	Factor ^4^	Percentage ^5^ [%]
2	4-ethylguaiacol	44	952	22	5 // 95
3	4-vinylguaiacol	290	7890	27	4 // 96
4	*(E)*-β-damascenone	1.6	52	33	3 // 97
5	dimethyl trisulfide	52	855	16	6 // 94
6	guaiacol	34	22000	647	0 // 100
7	indole	52	33200	638	0 // 100
8	methional	82	904	11	9 // 91
9	skatole	28	97	3	29 // 71
10	vanillin	510	6300	12	8 // 92

^1^ Numbering is in accordance to online supplementary material Table S1; ^2^ As no quantitation of oct-1-en-3-one was possible in hydrolyzed urine (and the numbering is in accordance to online supplementary material Table S1), number 1 (oct-1-en-3-one) is left out in this table.; ^3^ Compounds listed in alphabetical order; ^4^ Factor: median concentration in glucuronidase-treated urine divided by median concentration in native urine; ^5^ Displayed is the average percentage of each compound present in its free form as opposed to its glucuronide conjugate, separated by a double slash.

The huge differences between native and glucuronidase-treated samples are also reflected when analyzing the data by statistics. The paired-sample Wilcoxon signed rank test revealed that the concentrations of dimethyl trisulfide, guaiacol, indole, methional, skatole, and vanillin were at *p* = 0.05 significantly higher in hydrolyzed than in native urine. Besides, the paired-sample Wilcoxon signed rank test could not be performed on the data of 4-ethylguaiacol, 4-vinylguaiacol, and (E)-β-damascenone because the sample size was too small, as discussed before. In order to be able to estimate the ratio of free to glucuronidated acid and thereby to be able to include the acids in Figure 3a, b, we conducted some additional experiments, where we quantified butanoic acid and 3-methylbutanoic acid in one native urine sample each. The concentration of butanoic acid was 35,100 µg/mol creatinine, while that of 3-methylbutanoic acid was 29,300 µg/mol creatinine. As these are only data of one urine sample we did not include them in any of the tables. Still, they are another indication that the ratios of the free to the corresponding glucuronidated odorants are higher for the acids than for the remaining compounds.

The increased concentrations after de-glucuronidation can be easily explained for skatole, vanillin, 4-ethylguaicol, 4-vinylguaiacol, indole guaiacol, butanoic acid and 3-methylbutanoic acid as those compounds all have functional groups which are known to be glucuronidated (in these cases amino, hydroxyl and carboxy groups) [57,58].

**Figure 4:**
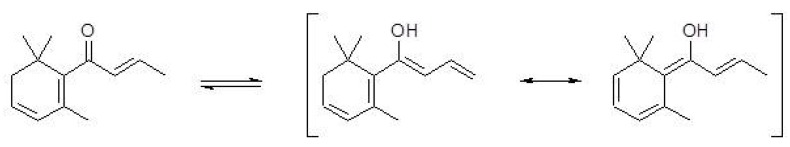
Keto-enol tautomerism of *(E)*-β-damascenone and resonance stabilization of the enol.

In contrast to this, the increased concentrations of *(E)*-β-damascenone, methional and dimethyl trisulfide is striking as one would not expect those compounds to be found as glucuronides when regarding their chemical formulas. The reason why *(E)*-β-damascenone is present in higher concentrations after enzymatic hydrolysis than in the native samples may be that it is in fact glucuronidated. At first sight, *(E)*-β-damascenone does not have the functional groups which are commonly required for glucuronidation. However, *(E)*-β-damascenone may form a hydroxyl group by keto-enol tautomerism (Figure 4). The resulting enolic form would be resonance-stabilized. Further, the equilibrium position of this tautomerism is of minor importance as the enol would be glucuronidated in the body and thus no longer be part of the equilibrium. According to Le Chatelier´s principle [59], further enol is formed and the equilibrium is constantly rebuilt. When subjecting the urine to enzymatic hydrolysis, the enol would be freed by de-glucuronidation and the ketone would be formed until equilibrium is reached.

Dimethyl trisulfide is an aroma compound present in substantial amounts in some foods like red and white cabbage [45]. It is formed by oxidation of the thiol methyl mercaptan and further disproportionation of the resulting dimethyl disulfide. Methyl mercaptan is formed from methional which originates from methionine by Strecker degradation [45]. Increased release of dimethyl trisulfide in the context of glucuronidase assays has, to the best of our knowledge, not been reported until today. Overall, the increased release processes of this substance as well as of others monitored in our study as for example methional will need further targeted investigations. In any case, one needs to keep in mind that these processes might relate not only to the pure enzymatic de-conjugation step but might additionally be superimposed by, e.g., oxidation reactions or other follow-up reactions with other (odorless) constituents that are present in the substance mix obtained after enzymatic treatment. Accordingly, additional studies need to be accomplished with more detailed focus on the underlying precursor substances to obtain a comprehensive picture of the relevant substances and their formation.

Based on the knowledge of common odorants and odorant conjugate profiles, further studies may then distinctly target, e.g., specific dietary influences on the urinary odorant profiles as they are quite evident for example in the case of asparagus [19,20]. Further, characteristic changes in urinary smell, and specifically the underlying molecular composition, due to diseases as discussed in the introduction represents another attractive area of research. Utilization of this important diagnostic mean, as already applied by our ancestors, e.g., in medieval times might thereby encounter a renaissance, supported by highly sensitive and selective analytical tools that are available nowadays.

## 4. Experimental

### 4.1. Chemicals

The following reference compounds were obtained from the suppliers shown: 3-(methylthio-) propanal (methional) ≥ 97%, 3-methylindole (skatole) ≥ 98%, 3-hydroxy-4,5-dimethyl-2(5*H*)-furanone (sotolone) ≥ 97%, 4-ethyl-2-methoxyphenol (4-ethylguaiacol) ≥ 9%, 2-methoxyphenol (guaiacol) ≥ 98%, 2-methoxy-4-vinylphenol (4-vinylguaicol) ≥ 98%, dimethyl trisulfide ≥ 98%, 3-methylbutanoic acid ≥ 99%, 2-methylbutanoic acid ≥ 98%, *(E)*-1-(2,6,6-trimethyl-1-cyclohexa-1,3-dienyl) but-2-en-1-one (*(E)*-β-damascenone) 1.1–1.3 wt. %, and 1-octen-3-one 50% solution in 1-octen-3-ol from Aldrich (Steinheim, Germany). 4-hydroxy-3-methoxybenzaldehyde (vanillin) ≥ 99% was obtained from ABCR (Karlsruhe, Germany). Indole ≥ 98.5% and butanoic acid ≥ 99.5% were purchased from Fluka (Steinheim, Germany). The following stable isotope labeled standards were from aromaLAB AG (Freising, Germany): [^2^H_3_]-1-octen-3-one, [^2^H_3_]-methional, [^13^C_2_]- sotolone, [^2^H_3-4_]- *(E)*-β-damascenone, [^2^H_6_]- dimethyl trisulfide, [^2^H_7_]- skatole, and [^2^H_3_]- 4-vinylguaiacol. The stable isotope labeled standards [2H_5_]-4-ethylguaiacol, [^2^H_3_]-guaiacol, 2,2-[^2^H_2_]-3-methylbutanoic acid, and [^2^H_7_]-indole were purchased from Dr. Ehrenstorfer / CDN-isotopes (Pointe-Claire, Quebec, Canada). [^13^C_6_]- Vanillin and [^13^C_2_]- butanoic acid were from Aldrich (Steinheim, Germany).

Dichloromethane p.a. and acetic acid ≥ 99.95% were obtained from Th. Geyer GmbH & Co. KG (Renningen, Germany), and sodium azide ≥ 99.5% as well as β-glucuronidase with < 7,500 unit/mL sulfatase activity from Helix pomatia type HP-2 (aqueous solution > 100,000 units/mL) were from Sigma (Steinheim, Germany).

### 4.2. Study Design

The study design was in concordance with the requirements of the declaration of Helsinki, and was conducted after consultation of the local ethical committee.

### 4.3. Donors

Donors were volunteers (8 females and 6 males, age range 22–36, mean age 28), exhibiting no known illnesses at the time of examination, and consuming their freely chosen meals without any specified dietary protocol but were asked to refrain from drinking coffee for two days before sample collections. The volunteers were asked to keep dietary records for two days before they provided the urine sample. Prior to sample collection and analysis written consent was obtained from all participants after a full explanation of the purpose and nature of the study.

### 4.4. Samples

Random urine samples [9] were collected in sterile amber glass bottles. The urine samples were processed and analyzed directly after donation as described below. To account for possible contamination and illnesses that probands were not aware of, dipstick-testing (multiproperty strips-testing) was carried out on all samples making sure that no urine with abnormal values was included in the experiments. Therefore, multiple test stripes “Combi-Screen PLUS” from Analyticon Biotechnologies AG (Lichtenfels, Germany) were used, providing the possibility to simultaneously test ascorbic acid, bilirubin, blood, glucose, ketones, leucocytes, nitrite, pH, protein, specific gravity/density and urobilinogen.

### 4.5. Measurement of Creatinine

Creatinine was measured for calculation of analyte / creatinine-ratios, in accordance with the generally approved correction of diuresis using urinary creatinine concentration [9]. Generally, measurement of creatinine is recommended by the European Confederation of Laboratory Medicine when quantitative determinations in urine are performed and timed collections overnight or for 24 h are to be avoided [9]. Accordingly, a method based on the reaction of Jaffé was used for creatinine measurement in our experiments using the creatinine kit from Labor + Technik Eberhard Lehmann GmbH (Berlin, Germany).

### 4.6. Solvent Extraction and Solvent Assisted Flavor Evaporation of Urine Volatiles

Solvent assisted flavor evaporation (SAFE) [60] was applied as described in our previous study [6] for the fast and careful isolation of the urinary volatiles. The urine was either subjected to the SAFE directly for isolation of the volatile fraction, or immediately subjected to the hydrolysis procedure with β-glucuronidase prior to SAFE as described below, and subsequently subjected to volatile isolation via SAFE.

#### 4.6.1. Isolation of the Volatile Fraction without Enzymatic Hydrolysis

The labeled internal standards dissolved in dichloromethane were added to 50 mL fresh urine. The standards were added in similar concentrations as present in urine based on precedent orienting trials. Then, 25 mL of freshly purified dichloromethane were added, the mixture was stirred for 30 min, and immediately applied for mild distillation at 50 °C. After distillation of the mixture additional aliquots of 10 mL of dichloromethane were administered and distillation was re-performed thrice to achieve complete transfer of the respective odor compounds. The obtained aqueous distillate phase was additionally extracted thrice with 50 mL of dichloromethane. Then all combined dichloromethane phases were dried over anhydrous Na_2_SO_4_, and finally concentrated to a total volume of 100–200 μL at 50 °C by means of Vigreux-distillation and micro-distillation [61]. Blank samples were prepared by subjecting only dichloromethane (same amount as added to the urine samples) to SAFE and treating the resulting distillate exactly like the urine samples.

#### 4.6.2. Enzymatic Hydrolysis (β-Glucuronidase Assays)

Exactly 10 mL of an acetic acid—sodium acetate-buffer adjusted to pH5 were added to 10 mL fresh urine in concordance with the methodology previously described in [6]. Then 0.2 mL of the β-glucuronidase-solution was added. The mixture was stirred for 15 h at 37 °C. Afterwards, 10 mL of purified dichloromethane and the labeled internal standards dissolved in dichloromethane were added and the mixture was stirred for 30 min.

Subsequently, the glucuronidase-treated samples were subjected to SAFE distillation analogous to the outline described above for the untreated samples. Therefore, additional aliquots of 5 mL of dichloromethane were administered and distillation was re-performed thrice. The distillate phase was dried over anhydrous Na_2_SO_4_, and finally concentrated to a total volume of 100–200 μL at 50 °C by means of Vigreux-distillation and micro-distillation.

Blank samples were prepared by adding the sodium acetate-buffer to highly purified water instead of urine and treating this mixture as described above for the urine samples. Blank samples were analyzed as described for the native urine as well as the glucuronidase-treated samples.

#### 4.6.3. Addition of Sodium Azide

As glucuronidase-assays were incubated for 15 h at 37 °C we conducted additional experiments to exclude the influence of bacterial growth in the course of the quantification experiments. Therefore, two urine samples were processed in two different ways each. One part of each of the two samples was processed precisely as described in Section 4.6.2. The second part was processed quite similarly, but 1 mL sodium azide solution (200 mg/mL in deionized water) was added to the urine samples immediately at the beginning of the experimental procedure.

### 4.7. Two-Dimensional High Resolution Gas Chromatography-Mass Spectrometry (TD-HRGC-MS)

A two-dimensional gas chromatographic system (2D-HRGC) was applied with the specifications given in [6]. Mass spectra were generated in positive CI mode (m/z range 35–249) with methanol as reagent gas. Ion source temperature was kept at 190 °C, emission current was 10 µA and ionization energy was 70 eV. The intensities of the selected ions of the odorants and the labeled standards were calculated by MS Data Review, Varian MS-Workstation (Version 6.9; Service Pack 1, Varian, Inc.). Calibration curves of defined mixtures of odorants and isotopic labeled standards (5:1, 3:1, 1:1, 1:3, 1:5, w/w) were measured and calibration functions were calculated by using the relative intensities of the respective mass ions. The concentration of an odorant in human urine was obtained by calculating the intensity of selected ions for the odorant as well as for the matching isotopic labeled standard, incorporating the results of the calibration function and the known amount of isotopic labeled standard added to the sample as described by [62]. Standard compounds, isotopic labeled standards, their mass, and the m/z selected for quantification are given in online supplementary material Table S2 for compounds quantified in native urine and Table S4 for compounds quantified in hydrolyzed urine samples.

### 4.8. High Resolution Gas Chromatography-Mass Spectrometry (HRGC-MS)

Quantification of the two acids butanoic acid and 3-methylbutanoic acid was carried out using a one-dimensional HRGC-MS system as the concentrations of the acids were high enough to quantify these substances without the need for two-dimensional gas chromatographic separation. Moreover, the acids are polar compounds, thus a better separation could be achieved using a polar column (DB-FFAP) only. The one-dimensional system was a Finnigan Trace GC Ultra (Thermo Electron Corporation/Thermo Scientific) coupled to a Thermo DSQ Single Quadrupole MS (Thermo Electron Corporation/Thermo Scientific). The approach was the same as described above (4.7) for the remaining compounds. The software was Xcalibur Data System (Version 1.4, Thermo Electron Corporation / Thermo Scientific).

### 4.9. Statistical Analyses

The Mann-Whitney U test was used for the comparison of concentrations of selected odorants in urine of female and of male test persons. The paired-sample Wilcoxon signed rank test was used for the comparison of concentrations of odorants in native and hydrolyzed urine samples.

## 5. Conclusions

Pronounced inter-individual differences in the excretion of both un-metabolized as well as glucuronidated substances were found for most of the odorants analyzed in this study. Moreover, pronounced gender-related differences could be observed in the excretion of un-metabolized odorants. Comparing the concentrations of the odorants in native and hydrolyzed urine, a distinct increase after deglucuronidation was observed for most compounds.

Generally, from our observations one can conclude that the investigation of odorants and volatiles/semi-volatiles in general obviously opens up a new window into physiological processes occurring in our organism. Further studies might also target at disturbances of these processes due to general physiological, hormonal or potential disease-related changes, and might bare the potential to serve as a diagnostic mean. There is a series of factors which may contribute to the variations observed in this study, for example differences in diet or life style (e.g., uptake of volatiles or odorants from smoking or hygienic products), metabolization and resorption processes. Further investigations will be needed to shed light on their relative contribution to volatile and odorant profiles in humans. Generally, our study shows that we are just on our way to understanding the presence and composition of volatile classes in our organism that have rarely been addressed until today. The diagnostic potential of odorless compounds might therefore be similar to that of odor-active compounds. Nevertheless, the latter are a group of substances that have to date been seldom addressed and which are often present at concentrations that elude detection when they are not specifically focusing on. On the other hand, urinary odor changes during the development of diseases have been shown to be of additional diagnostic value, potentially adding further specificity to the diagnostics. In this context, one needs to keep in mind that urinary excretion strictly relates to systemically circulating substances in our body. Our studies show that these compounds represent (a) compounds that may easily access diverse physiological targets (specifically in the case of the free compounds), (b) might be easily distributed by diverse routes within the human organism, and (c) represent a substance group that has to be indeed regarded as a substantial mean due to their obvious quantitative presence. Accordingly, future research in this field will further need to translate the knowledge about their presence in the human body into targeted studies on their potential physiological meaning.

## Supplementary Files

Supplementary File 1 Supplementary1 (DOCX, 31 KB)

Supplementary File 2 Supplementary2 (JPG, 46 KB)

Supplementary File 3 Supplementary3 (JPG, 73 KB)
