# Supplementary material for: Quantitative Determination of Common Urinary Odorants and Their Glucuronide Conjugates in Human Urine
Source: Metabolites. 2013 Aug 7;3(3):637–57. doi: 10.3390/metabo3030637 (PMC3901281; doi:10.3390/metabo3030637)
Supplement: Supplementary File 1 — Supplementary1 (DOCX, 31 KB) [file metabolites-03-00637-s001.docx]

Supplementary

**Online supplementary material** **Table S1.** ***Concentrations of selected odorants in native human urine.*** Displayed are the minimum, the maximum, and the median concentrations in the non-normalized form as well as normalized by creatinine concentration.

| **No.** ^1^ | **Compound** ^2^ | **Minimum concentration [µg/L]** | **Maximum concentration [µg/L]** | **Median concentration [µg/L]** | **Minimum concentration [µg/mol creatinine]** | **Maximum concentration [µg/mol creatinine]** | **Median concentration [µg/mol creatinine]** | **Number of samples** ^3^ |
| --- | --- | --- | --- | --- | --- | --- | --- | --- |
| 1 | oct-1-en-3-one | 0.03 | 0.13 | 0.08 | 1.32 | 34.5 | 10.4 | 5 // 7 |
| 2 | 4-ethylguaiacol | 0.04 | 1.47 | 0.26 | 4.39 | 386 | 43.9 | 7 // 8 |
| 3 | 4-vinylguaiacol | 0.62 | 2.80 | 1.06 | 42.9 | 501 | 290 | 5 // 4 |
| 4 | *(E)*-β-damascenone | 0.004 | 0.39 | 0.02 | 0.82 | 72.9 | 1.58 | 5 // 10 |
| 5 | dimethyl trisulfide | 0.08 | 1.02 | 0.20 | 8.99 | 177 | 52.4 | 6 // 0 |
| 6 | guaiacol | 0.04 | 0.54 | 0.13 | 4.29 | 96.2 | 33.6 | 5 // 5 |
| 7 | indole | 0.24 | 0.40 | 0.34 | 14.1 | 158 | 51.6 | 7 // 2 |
| 8 | methional | 0.26 | 1.34 | 0.51 | 32.5 | 239 | 82.3 | 8 // 2 |
| 9 | skatole | 0.06 | 0.30 | 0.10 | 4.79 | 60.3 | 27.8 | 9 // 0 |
| 10 | vanillin | 1.60 | 5.64 | 2.55 | 125 | 1,230 | 510 | 9 // 1 |

^1.^Numbering in accordance to online supplementary material Table S2; ^2.^Compounds listed in alphabetical order; ^3.^Determinations yielding values below or above the limit of quantification are separated from values within the calibration line by a double slash.

**Online supplementary material Table S2.** Selected ions, internal standards and chemical information on odorants in native human urine quantified by stable isotope dilution assays.

| **No.** ^1^ | **Compound** ^2^ | **Chemical formula** | **mass** | **Ion (m/z)** | **Internal standard** | **mass** | **Ion (m/z)** |
| --- | --- | --- | --- | --- | --- | --- | --- |
| 1 | oct-1-en-3-one | C_8_H_14_O | 126 | 127 | [^2^H_3_]-oct-1-en-3-one | 129 | 130 |
| 2 | 4-ethylguaiacol | C_9_H_12_O_2_ | 152 | 153 | [^2^H_5_]- 4-ethylguaiacol | 157 | 158 |
| 3 | 4-vinylguaiacol | C_9_H_10_O_2_ | 150 | 151 | [^2^H_3_]- 4-vinylguaiacol | 153 | 154 |
| 4 | *(E)*-β-damascenone | C_13_H_18_O | 190 | 191 | [^2^H_3-4_]-*(E)*-β-damascenone | 193–194 | 195 |
| 5 | dimethyl trisulfide | C_2_H_6_S_3_ | 126 | 127 | [^2^H_6_]- dimethyl trisulfide | 132 | 133 |
| 6 | guaiacol | C_7_H_8_O_2_ | 124 | 125 | [^2^H_3_]-guaiacol | 127 | 128 |
| 7 | indole | C_8_H_7_N | 117 | 118 | [^2^H_7_]-indole | 124 | 124 |
| 8 | methional | C_8_H_8_OS | 104 | 105 | [^2^H_3_]-methional | 107 | 108 |
| 9 | skatole | C_9_H_9_N | 131 | 132 | [^2^H_7_]- skatole | 138 | 139 |
| 10 | vanillin | C_8_H_8_O_3_ | 152 | 153 | [^13^C_6_]- vanillin | 158 | 159 |

^1^ Numbering in accordance to online supplementary material Table S1; ^2.^Compounds listed in alphabetical order.

**Online supplementary material Table S3.** ***Concentrations of selected odorants in glucuronidase-treated human urine.*** Displayed are the minimum, the maximum, and the median concentrations in the non-normalized form as well as normalized by creatinine concentration.

| **No.** ^1^ | **Compound** ^2^ | **Minimum concentration**  **[µg/L]** | **Maximum concentration**  **[µg/L]** | **Median concentration**  **[µg/L]** | **Minimum concentration**  **[µg/mol creatinine]** | **Maximum concentration**  **[µg/mol creatinine]** | **Median concentration**  **[µg/mol creatinine]** | **Number of samples** ^3^ |
| --- | --- | --- | --- | --- | --- | --- | --- | --- |
| 1 | 3-methylbutanoic acid | 569 | 9,560 | 1,050 | 38,700 | 1,800,000 | 174,000 | 12 // 0 |
| 2 | 4-ethylguaiacol | 3.53 | 77.0 | 6.88 | 322 | 8,760 | 952 | 5 // 6 |
| 3 | 4-vinylguaiacol | 16.3 | 171 | 46.9 | 1,000 | 89,000 | 7,890 | 8 // 6 |
| 4 | *(E)*-β-damascenone | 0.26 | 0.47 | 0.39 | 25.2 | 72.9 | 51.9 | 3 // 12 |
| 5 | butanoic acid | 272 | 784 | 346 | 32,000 | 168,000 | 61,100 | 11 // 1 |
| 6 | dimethyl trisulfide | 3.93 | 8.80 | 6.74 | 402 | 1,660 | 855 | 7 // 1 |
| 7 | guaiacol | 45.8 | 438 | 122 | 5,690 | 52,400 | 22,000 | 9 // 1 |
| 8 | indole | 34.1 | 287 | 186 | 13,400 | 49,100 | 33,200 | 7 // 2 |
| 9 | methional | 1.16 | 7.94 | 3.72 | 54.3 | 1,530 | 904 | 7 // 3 |
| 10 | skatole | 0.32 | 1.11 | 0.54 | 15.2 | 198 | 96.9 | 6 // 3 |
| 11 | sotolone | 31.5 | 434 | 88.1 | 5,990 | 226,000 | 10,800 | 4 // 11 |
| 12 | vanillin | 18.5 | 45.3 | 33.1 | 2,120 | 8,860 | 6,300 | 6 // 4 |

^.^Numbering in accordance to online supplementary material Table S4; ^2.^Compounds listed in alphabetical order; ^3.^Determinations yielding values below or above the limit of quantification are separated from values within the calibration line by a double slash.

**Online supplementary material Table S4.** Selected ions, internal standards and chemical information on odorants in glucuronidase-treated human urine quantified by stable isotope dilution assays.

| **No. ^1^** | **Compound ^2^** | **Chemical formula** | **mass** | **Ion (m/z)** | **Internal standard** | **mass** | **Ion (m/z)** |
| --- | --- | --- | --- | --- | --- | --- | --- |
| 1 | 3-methylbutanoic acid | C_5_H_10_O_2_ | 102 | 60 | 2,2-[^2^H_2_]-3-methylbutanoic acid | 104 | 62 |
| 2 | 4-ethylguaiacol | C_9_H_12_O_2_ | 152 | 153 | [^2^H_5_]- 4-ethylguaiacol | 157 | 158 |
| 3 | 4-vinylguaiacol | C_9_H_10_O_2_ | 150 | 151 | [^2^H_3_]- 4-vinylguaiacol | 153 | 154 |
| 4 | *(E)*-β-damascenone | C_13_H_18_O | 190 | 191 | [^2^H_3-4_]-*(E)*-β-damascenone | 193–194 | 195 |
| 5 | butanoic acid | C_4_H_8_O_2_ | 88 | 60 | [^13^C_2_]- butanoic acid | 90 | 62 |
| 6 | dimethyl trisulfide | C_2_H_6_S_3_ | 126 | 127 | [^2^H_6_]- dimethyl trisulfide | 132 | 133 |
| 7 | guaiacol | C_7_H_8_O_2_ | 124 | 125 | [^2^H_3_]-guaiacol | 127 | 128 |
| 8 | indole | C_8_H_7_N | 117 | 118 | [^2^H_7_]-indole | 124 | 124 |
| 9 | methional | C_8_H_8_OS | 104 | 105 | [^2^H_3_]-methional | 107 | 108 |
| 10 | skatole | C_9_H_9_N | 131 | 132 | [^2^H_7_]- skatole | 138 | 139 |
| 11 | sotolone | C_6_H_8_O_3_ | 128 | 129 | [^13^C_2_]- sotolone | 130 | 131 |
| 12 | vanillin | C_8_H_8_O_3_ | 152 | 153 | [^13^C_6_]- vanillin | 158 | 159 |

^1.^Numbering in accordance to online supplementary material Table S3; ^2.^Compounds listed in alphabetical order.
