# Supplementary figures and images for: Quantitative Determination of Common Urinary Odorants and Their Glucuronide Conjugates in Human Urine
Source: Metabolites. 2013 Aug 7;3(3):637–57. doi: 10.3390/metabo3030637 (PMC3901281; doi:10.3390/metabo3030637)

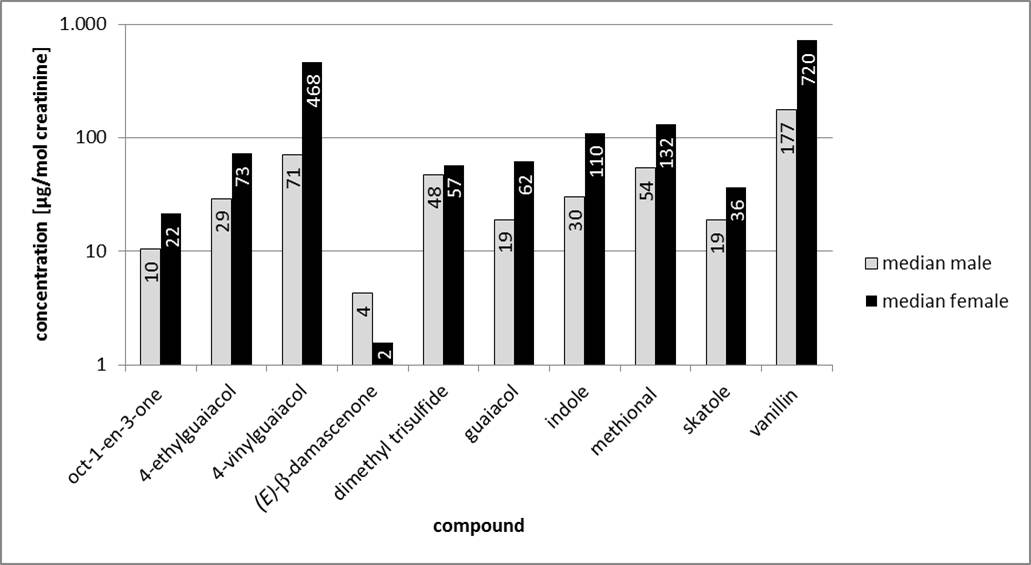

Supplement: Supplementary File 2 — Supplementary2 (JPG, 46 KB) [file metabolites-03-00637-s002.jpg]

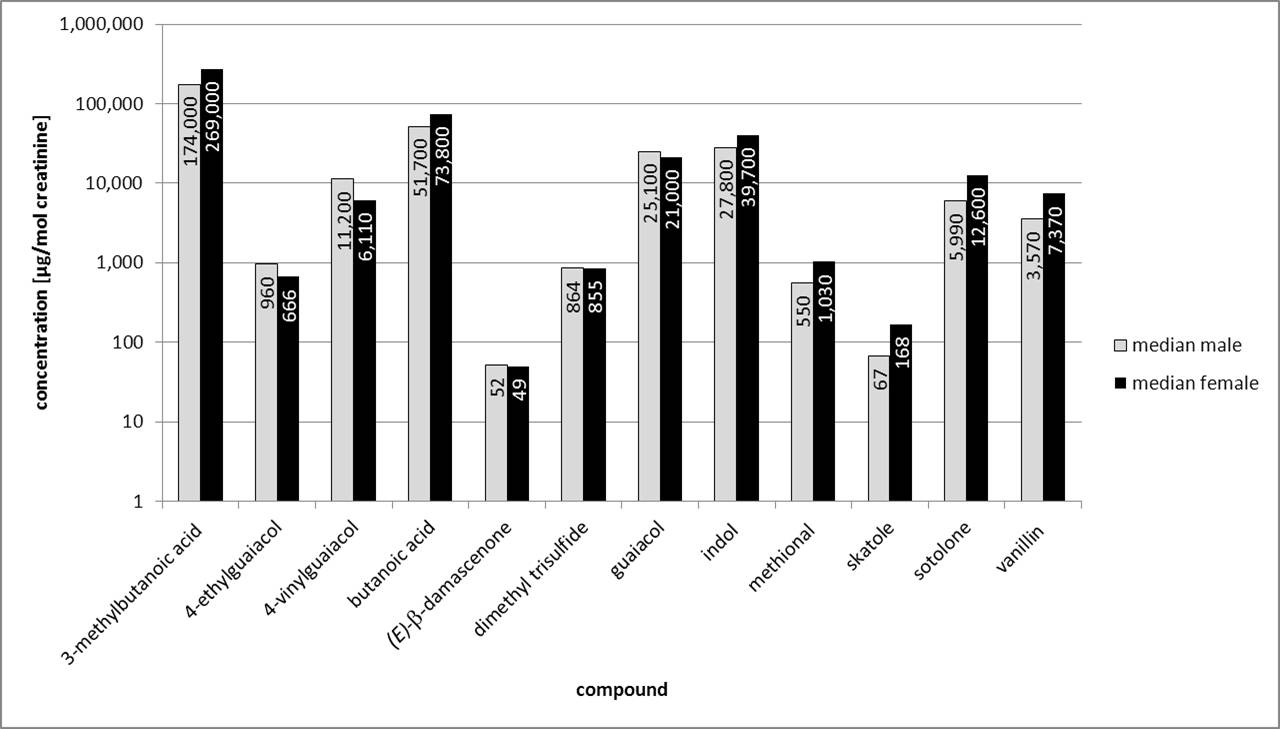

Supplement: Supplementary File 3 — Supplementary3 (JPG, 73 KB) [file metabolites-03-00637-s003.jpg]
